# Neocortical calretinin neurons in primates: increase in proportion and microcircuitry structure

**DOI:** 10.3389/fnana.2014.00103

**Published:** 2014-09-25

**Authors:** Domagoj Džaja, Ana Hladnik, Ivana Bičanić, Marija Baković, Zdravko Petanjek

**Affiliations:** ^1^Laboratory for Neuromorphometry, Department of Neuroscience, Croatian Institute for Brain Research, School of Medicine, University of ZagrebZagreb, Croatia; ^2^Department of Anatomy and Clinical Anatomy, School of Medicine, University of ZagrebZagreb, Croatia; ^3^Institute of Forensic Medicine and Criminalistics, School of Medicine, University of ZagrebZagreb, Croatia

**Keywords:** GABA, calretinin, neocortex, pyramidal neurons, species differences

## Abstract

In this article we first point at the expansion of associative cortical areas in primates, as well as at the intrinsic changes in the structure of the cortical column. There is a huge increase in proportion of glutamatergic cortical projecting neurons located in the upper cortical layers (II/III). Inside this group, a novel class of associative neurons becomes recognized for its growing necessity in both inter-areal and intra-areal columnar integration. Equally important to the changes in glutamatergic population, we found that literature data suggest a 50% increase in the proportion of neocortical GABAergic neurons between primates and rodents. This seems to be a result of increase in proportion of calretinin interneurons in layers II/III, population which in associative areas represents 15% of all neurons forming those layers. Evaluating data about functional properties of their connectivity we hypothesize that such an increase in proportion of calretinin interneurons might lead to supra-linear growth in memory capacity of the associative neocortical network. An open question is whether there are some new calretinin interneuron subtypes, which might substantially change micro-circuitry structure of the primate cerebral cortex.

## INTRODUCTION

**FIGURE 1:**
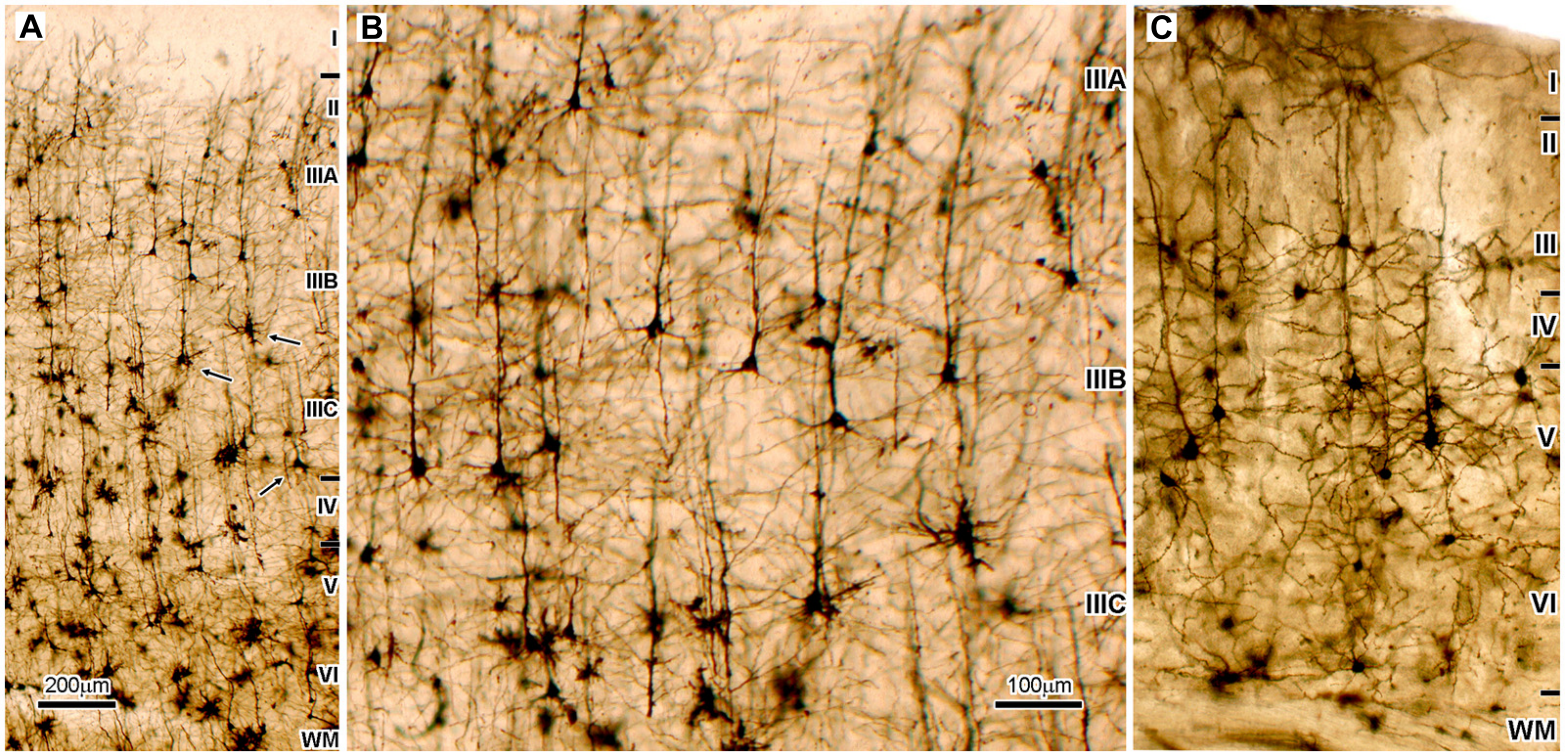
**Microphotography of the Golgi Cox impregnated sections of the associative areas in the human **(A,B)** and mice neocortex **(C)**. (A)** Dorso-lateral part of the frontal granular cortex (area 9) shows that supragranular cortical layers (II/III), which contain cortical projecting neurons, are two times thicker than infragranular layers (V/VI), which contain subcortical projecting neurons. In addition, pyramidal neurons (arrows) located deep inside layer III (sublayer IIIC) have largest cell body as well as most complex and extended dendritic arborization. Therefore they are the most prominent neurons found on Golgi staining, and on the Nissl staining they produce distinct cytoarchitectonic feature found only in high order associative areas of the cerebral cortex of human and apes, the magnocellularity (magnopyramidality) of the layer III (Petrides et al., 2012). Those neurons are on Nissl stained sections not only prominent by cell body size, they also have most intense cytoplasmatic staining showing high metabolic activity (Rajkowska and Goldman-Rakic, 1995a). They also have the most intense SMI32 staining, that indicates a very long and ramified axon tree (Morrison and Hof, 2002). The mentioned morphological features are a mark of associative cortico-cortical neurons. **(B)** Enlarged part of panel **(A)** is shown to be of the same magnification as panel **(C)**. **(C)** The highest order associative areas in the mice neocortex are located in the parieto-occipital region. When compared to highest order associative areas of the human neocortex **(A,B)** proportion of cortico-cortical projecting neurons is smaller than cortico-subcortical projecting neurons, and the largest neurons are layer V pyramids. Therefore, thickness of layers II/III in mice is less than half of the thickness of layers V/VI, which is opposite to human. In the parasensory associative areas (that do not have developed sublayer IIIC) of the human temporal cortex, layers II/III contain 44% of total number of neurons and have 30% more neurons than are located in layers V/VI (DeFelipe, 2011). It can be assumed that in areas with developed layer IIIC upper cortical layers contain more than 50% of neurons. In mice, upper cortical layers contain only 22% of neurons that is less than half of amount located in layers V/VI.

The main biological substrate for mammalian mental abilities is the neuronal circuitry of the cerebral cortex. Tremendous evolutionary increase in the neuron number and cortical connections (DeFelipe, 2011; Charvet and Finlay, 2012) allowed humans to adopt language and mathematical skills, to make affective modulation of emotional cues, possess self-conceptualization, mentalization, as well as to have high capacity of cognitive flexibility and working memory (Rakic, 2009). Such complex functioning is strongly related to distinct expansion of multimodal – high order associative areas, particularly the granular areas of the frontal lobe (i.e., associative prefrontal cortex; Teffer and Semendeferi, 2012). These areas have no clear correlate in mice and rats (Uylings and van Eden, 1990). In addition to expansion in size, there are significant changes in intrinsic organization of cortical circuitries (**Figure 1**). There are novel neuronal elements that appear in the human cerebral cortex making organization of microcircuitry (and consequently functional properties) substantially different when compared to non-primate mammals (Clowry et al., 2010).

In this article we first give a short overview of evolutionary changes in the connectivity of a specific class of principal (glutamatergic) cortico-cortical projecting neurons, as well as a possible functional significance of those changes regarding increase in cognitive capabilities. We also found that present comparative anatomical data suggest a distinct role in reorganization of cortical microcircuitry for one of the GABAergic local circuit neuron classes, the calretinin expressing neurons that in primates have much higher proportion. We propose a possible mechanism how calretinin neurons might contribute to reorganization of microcircuitry in the human associative cortex and how this might be related to an increase of cognitive capabilities.

## MICROCIRCUITRY CHANGES IN THE PRIMATE PREFRONTAL CORTEX AND INCREASE IN PROPORTION OF CALRETININ NEURONS

It is well recognized that upper layer pyramids (DeFelipe, 2011; Shepherd, 2011; Teffer and Semendeferi, 2012) are cortico-cortical projecting neurons (Elston et al., 2011; DeFelipe et al., 2012). It is less recognized that in primates, large deep layer III pyramids are long distance cortico-cortical neurons which establish in parallel connections with several cortical areas (Barbas et al., 2005; Yeterian et al., 2012). Experimental studies in rhesus monkey show that they are key elements in the circuitry involved in working memory and other prefrontal cortex-dependent associative cognitive functions (Wang et al., 2006; Verduzco-Flores et al., 2009). Data from various psychiatric disorders showed that selective alteration of large layer III pyramidal cells correlates with a decline in higher cognitive functions (Morrison and Hof, 2002; Selemon et al., 2003; Dean, 2009; Dorph-Petersen et al., 2009; Courchesne et al., 2011; Jacot-Descombes et al., 2012; Teffer and Semendeferi, 2012; Selemon et al., 2013) and developmental studies found that prominence in size of neurons in the upper cortical layers and peak in synaptic number appear by the end of infancy, stage when human specific mental capacities appear (Petanjek et al., 2008, 2011). Altogether, it can be concluded that large layer III pyramidal neurons in the high order associative areas are the main integrative elements (“associative” neurons) between different cortical areas (Goldman-Rakic, 1999; see discussion from Petanjek et al., 2008).

Pyramidal neurons located in upper layers of the primate prefrontal cortex also provide rich intracortical projections. From large layer III pyramids 80% of synaptic output belongs to local connections coming from axonal side branches (Melchitzky and Lewis, 2003). They extend several millimeters around, with dense columnar termination through layers II and III.

Human brain evolution is characterized by an increase in the number and width of minicolumns, but also in the increase of space available for interconnectivity between neurons, especially in the human prefrontal cortex where associative layer III pyramidal neurons are particularly abundant (DeFelipe et al., 2012; Spocter et al., 2012). Except to primary regions, prefrontal cortex established connections with all other cortical areas (Groenewegen and Uylings, 2000). That way efficacy of inter- and intra-areal integration within prefrontal cortex correlates with overall level of information processing, influencing consequently level of individual cognitive capability (Petanjek and Kostovic, 2012). Inter-individual differences in internal structure of upper cortical layers of human prefrontal cortex (Rajkowska and Goldman-Rakic, 1995a,b) make additional support that associative layer III neuron class has the major role in increasing the efficiency of cortico-cortical network (Buckner and Krienen, 2013; Hofman, 2014).

In parallel with evolutionary changes in connectivity of cortico-cortical network, significant changes appear in the organization of GABAergic network. This network acts as intrinsic modulator of cortical output since it is composed of local circuit neurons (interneurons; DeFelipe et al., 2013). Numerous studies analyzing laminar distribution and density of cortical GABAergic neuron subpopulations were performed in various species (**Table 1A**). It is curious that only rarely the same group performed a systematic analysis of several species, using the same methodology, making it difficult to conclude about interspecies differences. Most of the studies performed in rat and mouse found that cortical GABAergic neurons represent around 15% (14–16%) of the overall population. In monkey and human their proportion mostly exceeds 20% (20–29%) suggesting an increase in proportion for about 30–50%.

**Table 1:** Overview of publications quantifying proportion of GABAergic and calretinin neurons in the neocortex of rodents and primates. **(A)** Proportion of GABAergic cells in population of all neurons, and **(B)** proportion of calretinin neurons inside the GABAergic population.

(A) Percentage of GABAergic cells in the total neuron population		
**Anatomical area**	**Rat and mouse – GABA in total**	**Monkey and human – GABA in total**

Primary visual area (V1)	15% (Beaulieu et al., 1994) – Rat	20.5% (Beaulieu et al., 1992) – Monkey
	14.5% (Meinecke and Peters, 1987) – Rat	15% (Fitzpatrick et al., 1987) – Monkey
	15% (Lin et al., 1986) – Rat	20% (Hendry et al., 1987) – Monkey
Primary somatic sensory area (S1)	14% (Micheva and Beaulieu, 1995) – Rat	20–29% (Jones et al., 1994) – S1 and primary motor area-Monkey
	25% (Ren et al., 1992) – Rat	
Frontal lobe	22% (Santana et al., 2004) – Rat	24.9% (Gabbott and Bacon (1996) – Monkey
	16% (Gabbott et al., 1997) – Rat	21.2% (Hornung and De Tribolet, 1994) – Human
Temporal lobe		37.7% (del Rio and DeFelipe, 1996) – only layers II and III-Human
Multiple lobe analysis	19.5% (Tamamaki et al., 2003) – Mouse	25% (Hendry et al., 1987)
	15% (Beaulieu, 1993) – Rat	

**(B) Percentage of calretinin expressing neurons within GABAergic population**		
**Anatomical area**	**Rat and mouse – calretinin in GABA**	**Monkey and human – calretinin in GABA**

Primary visual area (V1)	17% (Gonchar and Burkhalter, 1997) – Rat	20% (Yan et al., 1995) – Monkey
	24% (Gonchar et al., 2007) – Mouse	
Frontal lobe	16.1% (Uematsu et al., 2008) – Rat	28.6% (Zaitsev et al., 2005) – Monkey
	24.7%* (Gabbott et al., 1997) – Rat	28.8%* – Human 34.2%* – Monkey (Sherwood et al., 2004)
	18% (Kubota et al., 1994) – Rat	33.2–44.8%* (Gabbott and Bacon, 1996; Meskenaite, 1997; Melchitzky et al., 2005)* – Monkey
Temporal lobe		46.2% (del Rio and DeFelipe, 1996) – only layers II and III-Human
Multiple lobe analysis	18% (Xu et al., 2010) – Mouse	
	13.9% (Tamamaki et al., 2003) – Mouse	

*Values reported with an asterisk have been calculated from values presented in the original papers.*

This large increase in proportion of GABAergic neurons seems to be principally caused by increase in number of neurons containing calretinin. Another two main classes, those containing parvalbumin and somatostatin, do not show such a robust increase in proportion (Hladnik et al., 2014). In rodents the proportion of calretinin neurons among the total population of GABAergic neurons is between 16–18%, whereas in primate the proportion of calretinin reaches in some areas 35–40% (**Table 1B**). del Rio and DeFelipe (1996) have estimated that, within layer II and III of associative temporal cortex in the human, GABAergic neurons represent around 1/3 of the total number of neurons, and almost half of GABAergic neurons express calretinin. In addition, a recent study (Ma et al., 2013) suggests that, in the human and monkey, calretinin neurons are two times more numerous in the frontal and parietal cortical areas. Collectively all these data indicate that the evolution lead to an increase in calretinin proportion in the upper cortical layers of high order associative regions. Our preliminary observations, comparing orbital frontal cortex in the rat and complementary area 14 in the rhesus monkey, showed a four- to fivefold increase in the proportion of calretinin for the upper cortical layers, where calretinin neurons cover almost 15% of the total number of neurons (Džaja et al., 2014).

## FUNCTIONAL PROPERTIES OF CALRETININ NEURONS

For efficient functioning of the human cerebral cortex with its complex areal subdivision and increased number of cortical columns, there is a need for enhanced inter-areal and intra-areal integration (Sherwood et al., 2005; Hofman, 2014). Appearance of most likely, evolutionary new associative neurons (DeFelipe and Fariñas, 1992; Nieuwenhuys, 1994; Spruston, 2008) makes substantial changes in the organization of microcircuitry and allows higher level of network integration (Buckner and Krienen, 2013). Inside the primate cortico-cortical network there is also a fivefold increase in proportion of calretinin neurons and it is reasonable to ask how this changes the microcircuitry structure.

Based on electrophysiological properties, two main types of calretinin interneurons can be distinguished in rodents: accommodating and non-adapting non-fast spiking cells (Markram et al., 2004; Butt et al., 2005; Caputi et al., 2009). These electrophysiological features are correlated with expression of a group of membrane voltage gated proteins (Markram et al., 2004), the calretinin cluster (Toledo-Rodriguez et al., 2004; Schwaller, 2014).

Different types of calretinin neurons can be identified based on their morphological features, particularly on the postsynaptic domain targeted by their axon. Double bouquet cells have vertically oriented axons which project mainly to basal dendrites of pyramidal cells (del Rio and DeFelipe, 1995; Yanez et al., 2005), while bipolar (Peters and Kimerer, 1981) and bitufted cells (Jiang et al., 2013) project to the proximal and middle region of pyramidal cell’s apical dendrite. Therefore, calretinin neurons provide direct inhibition, although with a low connectivity rate of ∼10% (Caputi et al., 2009), on mid-proximal dendritic domain of pyramidal cell (i.e., proximal parts of apical and basal dendrites). In addition to this sparse connectivity with nearby pyramids, calretinin neurons provide strong innervation onto somatostatin neurons (Pfeffer et al., 2013) and other calretinin cells (Caputi et al., 2009). These somatostatin neurons are known for providing direct inhibition of pyramidal cell’s apical and basal dendrites (Wang et al., 2004; Jiang et al., 2013), as well as for providing an inhibitory influence on parvalbumin neurons (Pfeffer et al., 2013).

Parvalbumin neurons are mostly basket cells, which exert strong inhibitory control over pyramid’s soma (Markram et al., 2004; Pfeffer et al., 2013). Optogenetic activation of fast spiking parvalbumin cells induces gamma oscillations in nearby pyramids (Cardin et al., 2009) and, without parvalbumin activity, pyramids would continue to fire but without synchrony (Gulyas et al., 2010). In other words, parvalbumin basket cells phase-lock their target pyramids through hyperpolarization, after which pyramids undergo rebound, but short lived depolarization and fire in synchrony (Cobb et al., 1995). Elimination of inhibitory influences on parvalbumin basket cells prolongs their influence on pyramids (Pouille and Scanziani, 2004), and this is where the potential role of calretinin neurons could reside. By inhibiting somatostatin neurons (Pfeffer et al., 2013; Cauli et al., 2014) they could create a disinhibitory window for parvalbumin baskets. We hypothesize that this, by calretinin neurons provided disinhibition, might prolong the effect of parvalbumin cells on pyramids, allowing longer periods of synchronized gamma oscillations.

This group of cells, including calretinin neurons, their somatostatin targets, parvalbumin neurons, and their pyramidal targets, can be collectively called a neuronal assembly (Borgers et al., 2012; Somogyi et al., 2014). The significance of an assembly is that it can activate its efferent targets with high probability, through the synchronous activity of its pyramids (Harris et al., 2003; Buzsaki and Wang, 2012). Interneurons are needed to segregate, maintain and also establish a temporal sequence of activation between particular assemblies (Somogyi et al., 2014). We hypothesize that the role of increased proportion of calretinin neurons would depend on the criteria of their territorial exclusivity, i.e., their developmental positioning to efferent targets compared to other calretinin interneurons. If newly added calretinin cells show no territorial exclusivity, i.e., their connections significantly overlap with those of pre-existent calretinin neurons, they would be incorporated into already established assemblies, connecting to somatostatin neurons to which some calretinin neurons have previously connected. Hence, there would be an increased number of calretinin cells per assembly, while the number of pyramids would not change, allowing for a more potent overall effect of calretinin neurons. However, in case of the other extreme, newly added calretinin cells will mostly connect to their own group of pyramids and parvalbumin and somatostatin neurons, creating smaller assemblies and allowing for more parallel processing. More parallel units might lead to supra-linear growth in memory capacity of the neocortical network, which is of particular importance in regions involved in planning and executive functions.

Present evidence suggests that the evolutionary path of the primate cortico-cortical network seems to have been an expansion in two aspects. First, there is an increase in proportion of principal neurons located in layers II and III, which would be a way to create the basic excitatory architecture for inter-areal processing. Second, there is an increase in proportion of calretinin expressing GABAergic interneurons, which would be a way to create a gain in synchrony and parallel processing between disparate cortical areas. An open question is whether this jump in proportion of calretinin neurons is based on a simple expansion of already preexistent subtypes of these cells found in the rodents or do we have some new cellular subtypes. If so, this might produce a more profound changes then simple supra-linear increase in their number, similar to changes occurring with appearance of associative principal neurons. These two might have been converging processes, making structure of microcircuitry in the primate neocortex substantially different when compared to other non-primate mammals.

## Conflict of Interest Statement

The authors declare that the research was conducted in the absence of any commercial or financial relationships that could be construed as a potential conflict of interest.
